# Promising landscape for regulating macrophage polarization: epigenetic viewpoint

**DOI:** 10.18632/oncotarget.17027

**Published:** 2017-04-11

**Authors:** Dexi Zhou, Kui Yang, Lu Chen, Wen Zhang, Zhenyu Xu, Jian Zuo, Hui Jiang, Jiajie Luan

**Affiliations:** ^1^ Laboratory of Clinical Pharmacy of Wannan Medical College, Wuhu, Anhui Province, China; ^2^ Department of Pharmacy in Yijishan Hospital of Wannan Medical College, Wuhu, Anhui Province, China

**Keywords:** macrophage polarization, microRNA, methlylation, histone modification

## Abstract

Macrophages are critical myeloid cells with the hallmark of phenotypic heterogeneity and functional plasticity. Macrophages phenotypes are commonly described as classically-activated M1 and alternatively-activated M2 macrophages which play an essential role in the tissues homeostasis and diseases pathogenesis. Alternations of macrophage polarization and function states require precise regulation of target-gene expression. Emerging data demonstrate that epigenetic mechanisms and transcriptional factors are becoming increasingly appreciated in the orchestration of macrophage polarization in response to local environmental signals. This review is to focus on the advanced concepts of epigenetics changes involved with the macrophage polarization, including microRNAs, DNA methylation and histone modification, which are responsible for the altered cellular signaling and signature genes expression during M1 or M2 polarization. Eventually, the persistent investigation and understanding of epigenetic mechanisms in tissue macrophage polarization and function will enhance the potential to develop novel therapeutic targets for various diseases.

## INTRODUCTION

Monocyte-macrophage lineage derives from myeloid precursors in bone marrow and subsequently develop as tissues-specific macrophages in response to local microenvironment signals [1, 2]. Macrophages are heterogeneous and pleiotropic cells which can be generally polarized into M1-(classically activated) or M2-(alternatively activated) subtypes, which is a continuum of diverse functional states [3, 4]. M1 phenotype could be triggered by lipopolysaccharide (LPS) and/or interferon (IFN)-γ andis believed to exert pro-inflammatory effects on tissue injury [5, 6], which have the specific markers such as inducible NO synthesase (iNOS), interleukin(IL)-12 [7, 8]. In contrast, M2 macrophages are known to be polarized by IL-4 or IL-13(M2a), immune complexes (M2b), or by glucocorticoids and transforming growth factor (TGF)-β (M2c), which could produce the M2 genes, such as genes chitinase-like protein (Ym1), found in inflammatory zone 1(Fizz1), arginase-1(Arg)-1, IL-10 and TGF-β [9–11]. There is a documented role for M2 in both wound healing and tissue remodeling by releasing a set of anti-inflammatory products [12]. The acquisition and maintenance of macrophage M1 or M2 phenotypes in diverse diseasesdepend on various signaling molecules and pathways controlled at transcriptional and post transcriptional levels [13, 14]., Additionally, there is growing evidence showing epigenetic modifications are involved in the macrophage polarization and function partially through the mechanisms, for example transcription inhibition and chromatin remodeling [15, 16].

MicroRNAs(miRNAs), DNA methylation(DNAm) and histone modifications have been reported as the best-known epigenetic markers and events in different regulatory networks. Firstly, miRNAs are defined as the short non-coding RNAs(ncRNAs) with about 22 nucleotides, which could posttranscriptionally lead the target-gene silencing by targeting the 3′untranslated regions (UTRs) of complementary mRNAs [17, 18]. MiRNAs-mediated macrophage polarization is a highly conserved process and important in contributing to either M1 or M2 polarization for several pathophysiologically divergent diseases [19]. Previous studies highlights the specific roles of a miR-dependent approach to manipulate the inflammation and immunity by controlling the subtle adjustment of macrophage phenotypes balance [20, 21]. Notably, DNAm is one of the best studied epigenetic regulatory system and is generally associated with transcriptional silencing. DNAm is essential for chromatin-associated gene silencing which is linked to the functions of methylated CpG islands [22, 23]. More recent studies using pharmacological and genetical approaches identify that DNAm is also associated with alterations in expression of M1/M2 genes [24]. Furthermore, DNAm is believed to integrate aberrant miRNAs function into multi-type molecular processes and macrophage heterogeneity. For example, the hypomethylated CpG sites with aberrant miRNAs are associated with monocytes aging [25]. Additionally, DNA methyltransferases1 (DNMT1)-mediated suppressors of cytokines signaling 1(SOCS1) hypermethylation, which result in the enhancement of LPS-induced pro-inflammatory cytokines expression in macrophages [26].

Finally, histone modifications are mainly thought to be the crosstalk between transcription factors and chromatin-modifying enzymes, which could function as epigenetic markers of chromatin state correlated with gene activation and repression [27]. Methods for measuring histone modifcations have identified that they associate with a variety of macrophage biological processes, including. survival, differention and activation [28–30]. Remarkably, analysis of specific histone modifications demonstrates that they serve as the predictors of M1/M2 polarization through the positively and negatively regulate the M1/M2 gene expression.

In general, in this review we include insights from signature genes, cellular signals, particularly epigenetic changes including miRs, DNAm and histones modifications, to highlight the general features of these modifications in the regulation of macrophage polarization and function Here we conclude the properties of macrophage polarization and function in the context of transcriptionally and post-transcriptionally biological processes. Epigenetic factors that directly or indirectly regulate the macrophage polarization and function have been linked to many human diseases pathogenesis. Taken together, more and more evidence that epigenetic alterations are crucially molecular mechanisms in controlling macrophage heterogeneity and plasticity might provide novel therapeutic targets in the light of macrophage-based cellular approaches.

## MICRORNA

Multiple miRs have been shown to be important mediators of macrophage physiological and pathological events such as proliferation, differentiation, activation and apoptosis [31–33]. Further studies indicating the alterated miRs regulatemacrophage inflammatory phenotype by targeting cellular signaling, gene expression and morphological features of macrophages [34–36]. Thus, there is a great need to identify functional miRs and find the mechanisms underlying miRs-exerted macrophage polarized effects. In conclusion, here we demonstrate the potential miRs which play central roles in the disturbance of a delicate equilibrium between the M1 and M2 profiles. We will also describe the factors that drive miRs-associated polarization of macrophages towards classically and alternatively activated phenotypes.

M1-polarized macrophage is commonly described as the pro-inflammatory cell type, which exhibit potent microbicidal properties and promote Th1 responses.

Analysis of different stimuli (LPS, oxLDLand IFN-γ) has identified the inflammatory miRNA(inflamma-miR) profiling in M1 polarized microglia/macrophags [37, 38]. Recent studies have revealed that many miRs play crucial roles in fine-tuning the level of M1 genes expression in different dieases, such as inflammatory diseases, rheumatoid arthritis(RA) and tumors. The pretreatment of chronic alcohol consumption augmented LPS-induced miR-155 levels in macrophages via NF-κB and the increased miR-155 contributes to alcohol-induced elevation in TNF-α production [39]. More recently, miR-155 knockout (KO) mice exhibited predominance of M2 phenotype when received alcohol diet for several weeks, which led to the decreased steatosis and inflammation in liver [40]. Elevated miR-155 levels are also the biomarkerof atherosclerotic lesions [41]. MiR-155 plays a pro-atherogenic role by promoting the SOCS1-STAT3-PDCD4 axis and expression of CC-chemokine ligand 2(CCL2) in M1-type macrophages, thereby enhancing vascular inflammation, plaque formation and rupture [42]. Silencing of the miR-155 target gene B-cell CLL/lymphoma 6 (Bcl6) in mice harboring miR155^−^/^−^ macrophages enhanced atherosclerotic plaques, and experiments performed by moxLDL/IFN-γ induction *in vitro* also confirmed it [43]. Bcl6 is therefore performed as the possible antiatherosclerotic targets through the intervention of miR-155 induced M1 polarization [43, 44]. Akt1 and Akt2 conversely mediated M1 and M2 polarization with the involvement of miR-155 [45–47]. The Akt1 suppression by miR-342-5p induces proinflammatory cytokines such as IL-6 in macrophages via the upregulation of miR-155 [47]. Thus, the crosstalk of miR-342-5p and miR-155 may offer a promising strategy to treat atherosclerotic vascular disease. MiR-155 in macrophages could possibly lead to the hierarchical miRs expression at least partially due to inhibition of the transcription factor CCAAT/enhancer-binding transactivator proteins(C/EBPβ) [48]. Both miR-155 and miR-146a are coordinately regulated the development of endotoxin tolerance via gene colocalization of C/EBPβ, NF-κB with the transcriptional machinery and histone3 methylation, in macrophages [49]. Synovial membrane and synovial fluid (SF) macrophages from patients with RA display up-regulated expression of miR-155,which lead to the inhibition of Src homology 2-containing inositol phosphatase-1 (SHIP-1) in CD68^+^ cells [50]. In turn, miR-155 inhibits the expression of SOCS1 and may lead to the upregulation of proinflmmatory cytokines (TNF-α and IL-1β) in macrophages of RA patients [51]. The upregulation of Notch-mediated miR-223 inhibits the aryl hydrocarbon receptor (AHR) signaling activation in CD14^+^macrophages and increases pro-inflammatory cytokines pruduction in RA [52]. Therefore, divergent miRs--based macrophage activation and polarization may be an intriguingly therapeutic target for RA. MicroRNA-155 promotes the pathogenesis of experimental colitis through the pro-inflammatory secretions including IL-6, TNF-α, IL-1β, and IFN-γ by repressing SHIP-1 expression [53]. Most importantly, miR-155 promotes the phenotypic skewing from M2 to M1 could by targeting M2-associated genes instead of M1-like genes [54], including lead to the inhibition of STAT6 by targeting IL-13Rα1 [55] and directly repress SMAD2 expression which influence TGF-β/Smad signaling pathway in the macrophage [56]. However, miR-155 a typical multifunctional microRNA which in atherosclerosis(AS) also acts as an anti-inflammatory microRNA [57], is evidenced by hematopoietic deficiency of miR155 enhances the ‘inflammatory’ monocyte subset (CD11b^+^Ly6G^−^Ly6C^hi^) and inhibits ‘resident’ monocytes (CD11b^+^Ly6G^−^Ly6C^low^) in the circulation [58]These results might be explained by the appropriate activation of miR-155 is used to hold the balance between M1 and M2 macrophages in the disease pathogensis.

**Figure 1:**
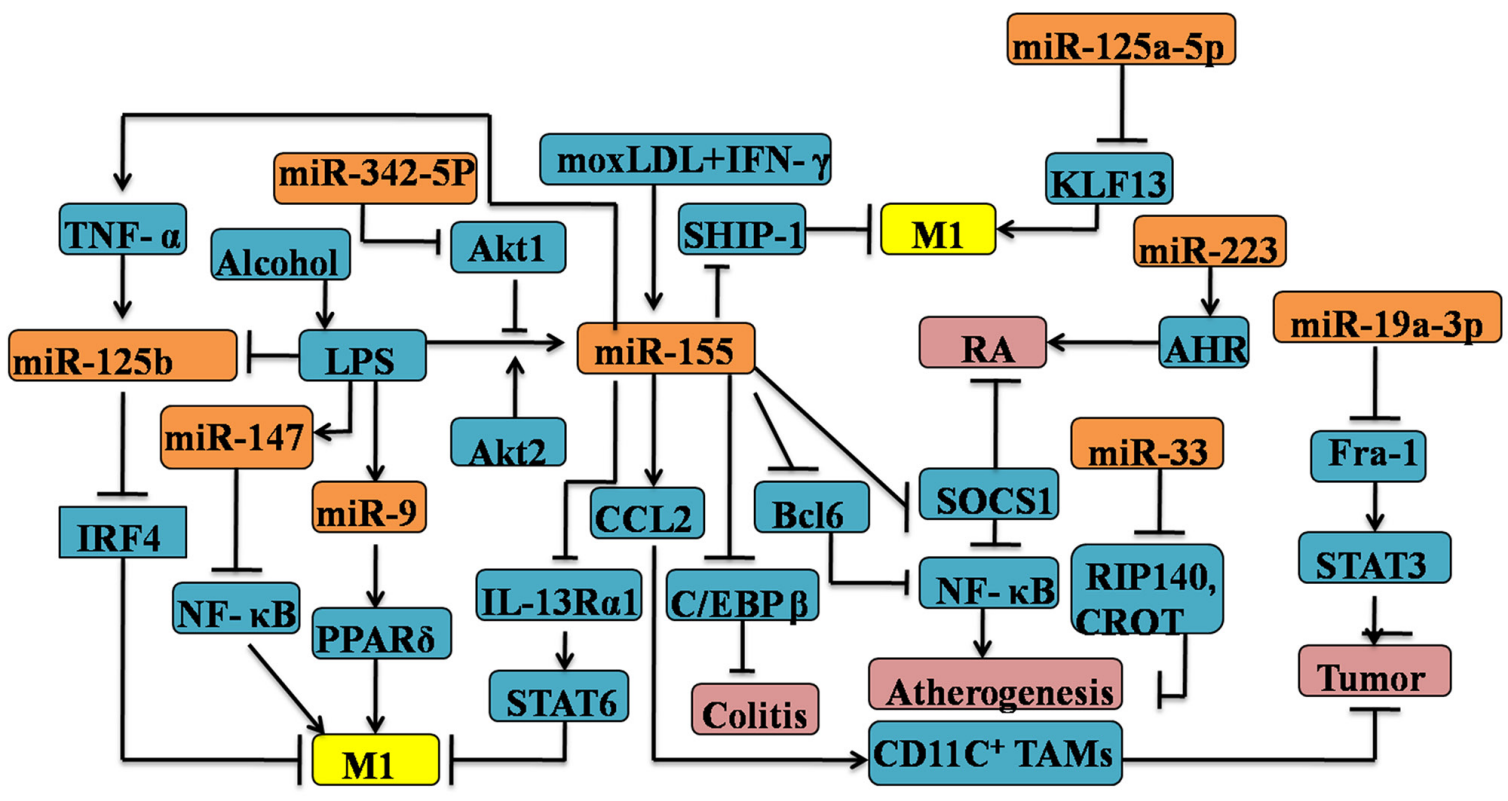
Regulation of M1 polarization by microRNAs in the macrophages Several mircoRNAs, including miR-9, 19a-3p, 33, 125a-5p, 125b, 146a, 147, 155, 223, 342-5p are involved in the M1 macrophage polarization by targeting the expression of various signature genes, such as C/REPβ, SOCS-1, NF-κB. MicroRNA-mediated M1 polarization has significant implications for various diseases, for example colitis, atherogenesis and other inflammatory diseases.

Many other miRs are involved in the establishment of M1 polarization. Previously, it was uncovered that miR-147 is involved in a negative-feedback loop in which TLR stimulation induces miR-147 to prevent excessive inflammatory responses in macrophages [59]. Peroxisome proliferator-activated receptorδ (PPARδ) is regulated by miR-9 in primary human monocytes stimulated with LPS, which is of great importance of skewing inflammatory M1-subtype [60]. M1-like macrophage is not only correlated with inflammatory diseases responses, but also is capable of involving in the invasion, migration and resolution of carcinoma. Tumor-associated macrophages (TAMs)polarization associated with the tumorigenesis is strongly relied on the well-programmed process of TAMs phenotype switch from an anti-tumoral M1-like phenotype to a pro-tumoral M2-like phenotype in the tumor microenvironment [61, 62]. MiR-19a-3p was downregulated in RAW264.7 cells of the M2 phenotype in conditoned medium of 4T1 mouse breast tumor cells. Most importantly, overexpression of miR-19a-3p could switch the TAMs phenotype from M2 to M1 with the consequence of downregulation of Fra-1 and it downstream genes VEGF, STAT3 and pSTAT3 [63]. Recent data highlight the anti-tumoral function of miR-155 by reprogramming the TAMs into M1-phenotype by the Akt signaling which constraining carcinogenesis [64]. Adipose tissue from obese individuals has been shown that the elevated expression of miR-125b which is associated to increase M1 macrophage polarization via directly repressing interferon regulatory factor 4(IRF4) levels [65–67]. Surprisingly, mouse Raw 264.7 macrophages stimulated by LPS resulted in the upregulation of miR-155, but down-regulation of miR-125b levels which was accompanied by the proper TNF-αproduction [68]. Therefore, the miR-125b-mediated M1 polarization may be triggered by TNF-α [69]. In summary, further investigation remains necessary for the deeper understanding of macrophages acquire the M1 phenotype by specific miRs (see Figure 1).

Considerable evidence has suggested that different miRs are involved in the anti-inflammatory action via regulating immune cells functions and immune responses. Herein, we clarify the functions and mechanisms of M2-associated miRs in various physiological and pathological conditions.

MiR-146a has been implicated as an essential molecule links the anti-inflammatory M2 polarization in different diseases. In systemic juvenile idiopathic arthritis (SJIA), knockdown of miR-146a promoted M1 but diminished M2 SJIA monocytes polarization by targeting INHBA [70]. Systematic studies of miR-146a functions in TAMs in breast cancer found that miR-146a enhanced the M2 molecules production and the antagomir of miR-146a transfected RAW264.7 cells inhibited 4T1 tumor growth [14]. Conversely, miR-222 in TAMs suppressed 4T1 tumor growth by downregulating CXCL12 and CXCR4. Notch1 signaling is also proved to be the target of miR-146a, which promote the M2 polarization of RAW264.7 cells [71]. Akt2 suppression and miR-146a induction skew the M2 phenotype by repressing expression of IRAK-1 and TRAF-6, resulting in the attenuation of lung injury induced by acid and LPS [72, 73]. Tumor cells migration and invasion can be largely diminished by miR-181a, which promote the M2 macrophages polarization through directly target KLF6 and C/EBPα [74]. TAMs are now considered to promote tumor progression in multiple ways and the miRs-mediated TAMs polarization might be necessary for the poor prognosis in tumor microenvironment. Let-7c is one of the first noted miRs, which expression is increased in alveolar macrophages from fibrotic lungs than controls and play pro-fibrogenic roles in lung. Further study demonstrate that let-7c and miR-125a-5p are expressed at a higher level in M-BMM (M2 macrophages) than in GM-BMM (M1 macrophages) and interestingly its level could be inversely changed when M-BMM converted to GM-BMM by targeting C/EBP-δ and KLF13 [75, 76]. Alcohol-exposed monocytes can stimulate naive monocytes to polarize into M2 macrophages via extracellular vesicles (EVs) and miR-27a cargo [77]. High levels of miR-27a in circulating EVs from plasma might be a potential therapeutic target for alcoholic hepatitis patients. Adipose tissue inflammation and systemic insulin resistance are more severer in miR-223^−^/^−^ mice, which might partially due to miR-223 could suppress M1 and in favor of M2 polarization pathway in macrophages by inhibiting Pknox [78]. An increased ratio of M1/M2 type markers in spinal cord microglia/macrophages is associated with persistent hyperalgesia in GRK2^+^/^−^ mice and reduced spinal cord microglia miR-124 levels. Then it was found that miR-124 treatment could restore the M1/M2 balance and reversed the persistent hyperalgesia by skewing the M2-like polarization via inhibiting the activation of C/EBP-α [79]. More interestingly, brain-specific miR-124 is expressed in microglia but not in peripheral macrophages, which directly inhibited the C/EBPα and its downstream target PU.1, could lead to the activated phenotype into a quiescent CD45^low^ MHC class II^low^ phenotype resembling resting microglia and suppress the experimental autoimmune encephalomyelitis (EAE) [31]. The anti-inflammatory functions of miR-181a were investigated in LPS-induced Raw264.7, the results indicated that miR-181a mimics significantly inhibited levels of inflammatory factors (IL-1β, IL-6, and TNF-α) at least in part by down-regulating IL-1α levels [80].

**Figure 2:**
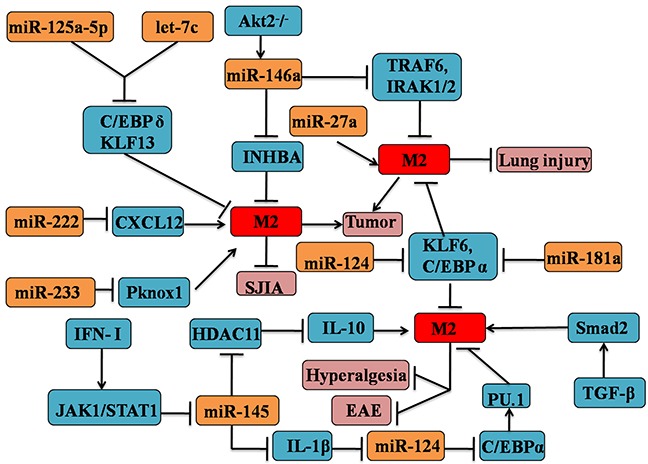
MicroRNAs are involved in the regulation of M2 macrophages phenotypic and functional polarization MicroRNAs (inculding let-7c, miR-27a, 124,145, 146a, 155, 181a, 124, 222 and 233) could contribute to the M2 macrophage polarization. MicroRNA-induced M2 polarization mainly depends on the interaction with cytokines signaling and transcription factors pathway, such as TGF-β, IL-10, STAT1, C/EBPα.

Increasingly studies show that the interaction of miRs and other epigenetic factors are now emerging as critical regulators in immune responses [81]. A study from Lin *et al*. firmly demonstrated that the activation of type I IFN (IFN-I) and downstream IFN-I receptor-JAK1-STAT1 signal cascade could inhibit the expression of miR-145 in macrophages, which through directly targeting the epigenetic IL-10 gene silencer histone deacetylase 11(HDAC11) [82]. In consequence, the enhanced IL-10 production lead to the suppression of inflammatory response, which might skew the macrophage to M2 phenotype [83, 84]. The roles of other epigenetic factors in the macrophage polarization will be discussed later in this review (see Figure 2).

## DNA METHYLATION

The aberrant occurrence of DNAm patterns (chemical modifications to the cytosine residues of DNA) has a significant influence on the biological behavior of macrophages [85, 86]. This part was therefore conducted to summarize that alterations in DNAm profiles, including both hyper- and hypomethylation, with the specific emphasis on the influence present in the macrophage polarization.

The Kruppel-like transcription factor (KLF) family participates in the activation and inflammation of myeloid and lymphocyte cell lineage during immune responses [87]. KLFs have been recognized as the molecular toggles controlling macrophage polarization, including KLF6 and 10 [88, 89]. KLF4 exhibits remarkable function to cooperate with STAT6 to induce an M2 genetic profile and inhibit M1-type targets via sequestration of coactivators for required for NF-κB activation [90]. More importantly, many studies recently proposed the association between KLF4 and DNAm [91, 92]. It was involved a common principle of recognition of methylated CpG by C2H2 zinc finger (ZnF) proteins, involving a spatially conserved Arg-Glu pair, which might be regulated by DNMT3a [91, 93]. Mechanically, KLFs are epigenetically involved in the macrophage polarization through the regulation of miRs and DNAm.

**Figure 3:**
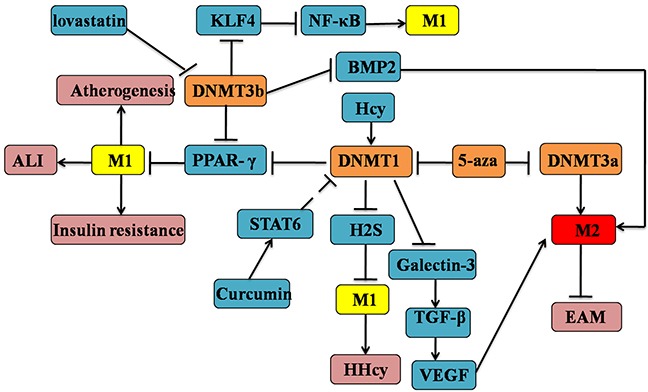
DNA methylation in the determination of macrophages M1/M2 polarization Firstly, DNMTs are responsible for catalyzing epigenetic silencing and inappropriate activation of gene expression involved with the macrophage phenotypic changes. Then, DNMTs (including DNMT1, 3a and 3b) are differentially expressed in M1 or M2 macrophages, which might play opposite roles in the M1/M2 polarization. For example, the activation of DNMT1/3 might lead to the M1 polarization by targeting KLF4 and NF-κB signaling, which could be inhibited by 5-aza. Conversely, it inhibits the M2 macrophage polarization via the disturbance of the TGF-β and VEGF signaling. Among this complex process, STAT6 and other genes also participate in it.

DNMTs are responsible for catalyzing epigenetic silencing and inappropriate activation of gene expression of DNAm [94]. There is novel evidence to suggest that DNMT 1, 3a and b are differentially expressed in M2 compared to M1 macrophage, which are all associated with gene silencing [95]. DNMT1-mediated M1 polarization is causally linked to the development of AS by directly target the promoter of PPAR-γ in macrophages [86]. The promoter of PPAR-γ is binded to DNMT3b, which may contribute to the M1 adipose tissue macrophages (ATMs) polarization and inflammation [24]. Hyperhomocysteinemia (HHcy) is an independent risk factor associated with the AS and other cardiovascular events. Hcy inhibits cystathionine γ-lyase (CSE) expression and hydrogen sulfide (H2S) production by binding to the CSE promoter region through the increased DNMT1 expression and DNA hypermethylation, which may trigger the elevations of pro-inflammatory cytokines (TNF-α, IL-1β) in macrophages [96]. Lund *et al*. unequivocally observed that atherogenic lipoproteins (APOE) promote global DNA hypermethylation in monocyte which is the effective markers of AS [97]. Therefore, DNMT inhibition or knockdown could decrease the M1 polarization, which provides a novel strategy for the prevention and therapy of AS. In addition, treatment with DNMT inhibitor 5-Aza 2-deoxycytidine(Aza) promotes an anti-inflammatory M2 macrophage phenotype and attenuation of acute lung injury(ALI) [98]. Pharmacologically using 5-aza or genetically by DNMT1 deletion inhibits PPARγ promoter DNAm and promotes M2 macrophage activation, which protected from the obesity-induced inflammation and insulin resistance [99]. Curcumin ameliorates experimental autoimmune myocarditis (EAM) by activating STAT6 and inducing M2 polarization of macrophages through a possible way by which inhibits DNMT [100]. Consequently, these data clearly demonstrate that DNA hypermethylation of multiple genes serves as s a critical determinant of macrophage polarization, which contributes to the development of many inflammatory diseases. It is important to note that the expression of DNMT3a and DNMT3al are increased significantly in M2 compared to M1macrophages, which is dramatically associated with AMPK-signaling [101]. Conversely, DNMT3b was significantly lower in M2 vs M1 adipose macrophages. Moreover, up-regulation of galectin-3 production is the characteristic of M2-like macrophage [102]. Galectin-3 ablation in tumor stroma and parenchyma could induce the M1-like TAMs and decrease angiogenesis through disturbing the responses of macrophages to the interdependent VEGF and TGF-β1 signaling pathways [103]. Furthermore, through the DNA methylation analysis by the bisulfate genome sequencing method [104], the loss of galectin-3 of associated with its promoter methlation which could be inhibited by the treatment of 5-aza. Given the mentioned statements it is therefore might exist a negative feedback loop between M2-like macrophage and galectin-3. Dysregulated bone morphogenetic proteins (BMP) may also contribute to the macrophage polarization [105]. The initiation and progression of renal cell carcinoma (RCC) is promoted by BMP-6-mediated IL-10 expression, which regulates M2 polarization. Another BMP, BMP-2 is also involved in controlling M2 macrophage by the regulation of jumonji domain containing-3 (Jmjd3), a histone 3 Lys27 (H3K27) demethylase [106]. And lovastatin was shown to inhibit DNMT activity *in vitro*, resulting in the demethylation of the BMP2 promoter region [107, 108]. Some other interplay between DNA methylation and histone modification in the macrophage polarization will be discussed later in this review (see Figure 3).

## HISTONE MODIFICATION

Epigenetic traits are tightly regulated by the major epigenetic modification: histone proteins associated with DNA (histone modifications). Histones may undergo divergent epigenetic changes, including methylation, acetylation, phosphorylation, ubiquitylation and SUMOylation, which are often involved in establishing patterns of gene dysregulation associated with altered chromatin states, leading to gene activation and gene silencing in a host of diseases [109–111]. In this part, we particularly focus on the latest advances in the field of the histones modifications profiles associated with the regulation of macrophages polarization to M1 or M2 phenotypes.

Expressions of multiple genes encoding enzymes are responsible for catalyzing and modifying various histones post-translational modifications, such as methyltransferases, demethylases, acetyltransferases and deacetylases, which are differentially expressed in M1/M2 statuses [112, 113]. Emerging findings have suggested the existence of new regulatory epigenetic-based macrophage polarization mechanisms of histones modifications in inflammation and immune regulation. As the result of the devoid of tri-methylated H3K27 in IL-4-induced M2 macrophages, human CCL1 gene is selectively targeted by aryl hydrocarbon receptor (AhR) [114]. Pro-inflammatory cytokines (TNF-α and IL-6) promoters have a histone 3 lysine 4- and H3K36 dimethylation effect by the specific methyltransferase SET and MYND domain-containing 2 (Smyd2), which can lead to the decreased M1 polarization and NF-κB and ERK signaling [115]. Histone modification of increased levels of H3 at TNF-α gene locus was similarly concomitant with the activation of M1 polarization and M1-related chemokines and cytokines in the low-level laser therapy (LLLT) of human THP-1 monocytes [116]. Collectively, histones modifications of genes required for M1 or M2 polarization might have the therapeutic potential in various pathologies. For example, emodin seemingly to bidirectionally restore macrophage M1/M2 polarization and immune homeostasis by inhibiting the removal of H3K27 trimethylation (H3K27me3) and the addition of H3K27 acetylation (H3K27ac), respectively [117]. Ornithine decarboxylase (ODC) could inhibit gastric and colonic inflammation by the deletion of M1 macrophage responses, which accompanied by the decreased H3K4 monomethylation, H3K9 acetylation and increased H3K9 di/trimethylation in primary macrophages [113]. As early stated, Jmjd3 is indispensable for M2 macrophage polarization in response to helminth infection and chitin, which depend on the demethylase activity of Jmjd3, lead to the inhibition of trimethylation of H3K27 by targeting a key transcription factor, IRF 4 [106]. Of note, Jmjd3-IRF4 axis was also essential for M2 microglia polarization [118], which therefore play a pivotal role in the reprogramming and maintenance of microglia phenotypes that may contribute to the immune pathogenesis of Parkinson's disease (PD). In keep with these results, demethylation of H3K27me3 in the nuclear factor-activated T cells (NFAT) c1 (Nfatc1) gene locus by Jmjd3, playing important roles in NF-κB ligand (RANKL)-induced osteoclast differentiation [119]. Conversely, Jmjd3 is the transcription factor NF-κB-dependent induced in response to microbial stimuli, and Jmjd3 modifies the transcriptional output of LPS-activated macrophages in an H3K27 demethylation-independent manner [120].

Histone acetyltransferases (HATs) andHDACs mediate the acetylation and deacetylation for histone lysine, respectively. Histone H3 and H4 acetylation were found to be strongly associated with developmental stage of human monocytes and regulated the TNF-α promoter [121] Loss of HDAC function has been strongly linked to inhibition of several inflammatory diseases [122]. Monocytes polarized by IFN-γ increased histone H4 acetylation at the TNF-α promoter via the ERK and p38 mitogen-activated protein kinases (MAPKs) signaling pathyways, lead to the durable effects on the activation of transcription factor-2 (TF-2) and M1 polarization [123]. Noticeably, previous findings have identified that SOCS3 as a modulator of M2 macrophage polarization [124]. LPS-induced STAT-3 and MAPKs activation, including ERK1/2, JNK, p38 pathways, combination with the acetylation of histones H3 and H4 on the SOCS-3 promoter, eliciting critical roles in SOCS-3 expression, which provides for feedback attenuation of cytokine-induced immune and inflammatory responses in macrophages and microglia [125]. Histone acetylation-mediated SOCS-3 production might be involved in the macrophage polarized status. HDAC3-deficient macrophages were unable to activate almost half of the inflammatory gene expression profiles when stimulated with LPS, which resulted of decreased expression of IFN-β and Cox-1 [126]. Thus, the inhibitor of HDACs has a causative role in developing as anti-inflammatory agents. In consistent, M2-like macrophage activation is mediated by the HDAC3 by targeting PU.1 promoter region [127]. The macrophage lacking of HDAC3 displays a phenotype similar to IL-4 stimulation and thereby ameliorate many inflammatory diseases, such as pulmonary inflammation [127, 128].

**Figure 4:**
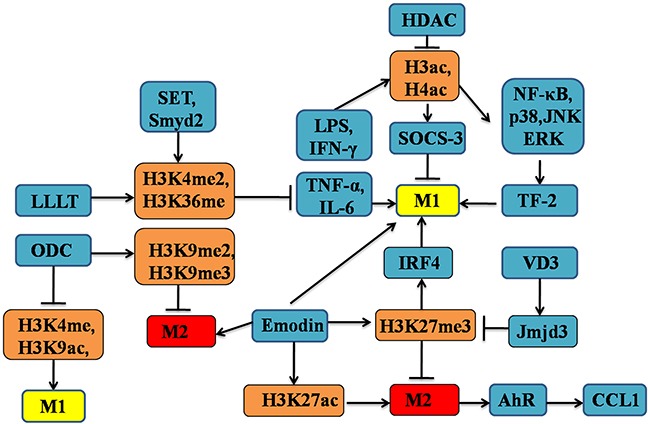
The altered patterns of histone modifications in the regulation of M1/M2 macrophages polarization Epigenetic regulation of histone modifications patterns, such as methylation, and acetylation, could drive macrophage polarization into either M1 or M2 phenotype. Of note, many genes are associated with histone modifications-dependent macrophage polarization, including Jmjd3 and MAPKs. These genes could cooperate with HDACs, HATs and other proteins, which exerting important effects on macrophage polarization and function.

Finally, secretion of some bioactive substances in a signal- and context-specific manner could influence macrophage polarization by targeting histone modifications [126]. For example, vitamin D3 (VD3) interacts with the signalling of transcription factors, also participates in the macrophage activation and polarization as the immunosuppressor [129, 130]. Most importantly, the VD3 exerts an ample regulatory effect on the expression of HDACs, such as Jmjd3, involved in epigenetic regulation that may mediate its actions on gene transcription and macrophage phenotype [131]. In summary, the relationships between histone modifications and macrophage polarization have significant implications for understanding the mosaic patterns of macrophage heterogeneity as well as function (see Figure 4).

## CONCLUDING REMARKS

Recent data highlight how some other epigenetic changes impact macrophage functional responses and M1/M2 polarization, influencing the immune homeostasis in response to infection and inflammation. Long non-coding RNAs(lncRNAs) expression profiles are significantly altered in macrophages exposure to differently incubated conditions, which evokes the distinct M1/M2 functional responses [132]. Of note, lncRNA MALAT1-overexpressed mesenchymal stem cells (MSCs) supernatants may serve to promote the M2 macrophage polarization, which may enhance the immunosuppressive properties of MSCs *in vivo* [133]. The crosstalk of lncRNA and other epigenetic manner could determine the macrophage phenotypes. SNHG14 promoted the microglia cells (MCs) classical activation by the inhibition of miR-145-5p, which resulted in the high levels of TNF-α and NO in MCs during cerebral infarction [134, 135]. In conclusion, the underlying epigenetic mechanisms of macrophage polarization and function might be indicative of functional association. Macrophage-dependent inflammatory responses are considered a pivotal biological process and remain to be fully elucidated, which contributes to fit the therapeutic requirement of antiviral, antibacterial or antitumor immunity in a host of diseases.

## Abbreviations

MAPKs: mitogen-activated protein kinases
miRs: microRNAs
DNAm: DNA methylation
ncRNAs: non-coding RNAs
UTRs: untranslated regions
PPAR: peroxisome proliferator-activated receptor
CCL: CC-chemokine ligand
Bcl6: B-cell CLL/lymphoma 6
STAT: signal transducers and activators of transcription
AP-1: activator protein-1
C/EBP: CAAT/enhancer-binding proteins
SOCS: suppressor of cytokine signaling
PBMC: peripheral blood monocytes
TLR: toll-like receptors
TAMs: tumor-associated macrophages
iNOS: inducible nitric oxide synthase
IRF: interferon regulatory factors
SJIA: juvenile idiopathic arthritis
EVs: extracellular vesicles
5-Aza: 5-Aza 2-deoxycytidine
EAE: experimental autoimmune encephalomyelitis
Ym1: geneschitinase-like protein
Fizz1: found in inflammatory zone 1
TGF-β: transforming growth factor-β
HHcy: hyperhomocysteinemia
CSE: cystathionine γ-lyase
H2S: hydrogen sulfide
DNMT: DNA methyltransferases
H3K27: histone 3 Lys27
TF: transcription factor
HATs: histone acetyltransferases
HDACs: histone deacetylases
RANKL: nuclear factor-kB ligand
ALI: acute lung injury
KLF: Krüppel-like transcription factor
Jmjd3: jumonji domain containing-3
LLLT: low-level laser therapy
ODC: ornithine decarboxylase
